# Serum tyrosine increases all-cause mortality in an older population

**DOI:** 10.3389/fendo.2025.1552752

**Published:** 2025-07-23

**Authors:** Yuhong Dai, Yong Zhang, Yue Zhang, Haoran Zheng, Ling Xiang, Liang Cheng, Xiaoqing Wang, Jie Zhang, Hairong Hao, De Huai, Wen Hu

**Affiliations:** ^1^ Department of Endocrinology and Metabolism, The Affiliated Huai′an Hospital of Xuzhou Medical University, Huai′an, China; ^2^ Department of Head and Neck Surgery, The Affiliated Huai′an Hospital of Xuzhou Medical University, Huai′an, China

**Keywords:** amino acids, tyrosine, all-cause mortality, China, older population

## Abstract

**Purpose:**

Amino acids play crucial roles in metabolic and cardiovascular diseases, especially branched-chain and aromatic amino acids, but their association with mortality remains understudied and inconclusive. This study explored the potential correlation between serum amino acids levels (including leucine(Leu), isoleucine(Ile), valine(Val), phenylalanine(Phe), and tyrosine (Tyr) and all-cause or cardiovascular deaths in an older population.

**Methods:**

This study involved 1,238 older people selected from the Huai’an Diabetes Prevention Program aged ≥ 60 years. Baseline serum levels of five amino acids (Leu, Ile, Val, Phe and Tyr) were measured. Participants were followed for 9 years. Cox regression analysis and Fine-Gray subdistribution hazard analysis were employed to assess the association between amino acids with all-cause or cardiovascular mortality. The prognostic value of amino acids was further assessed using the C index and Net Reclassification Improvement (NRI).

**Results:**

1 During the 9-year follow-up, 69 deaths occurred, including 32 from cardiovascular causes. Preliminary univariable analyses showed that only serum Tyr levels were associated with the risk of all-cause mortality among the five amino acids (per 1-μg/mL increase in Tyr, HR = 1.08, 95% CI = 1.01-1.17). 2 After adjustment for potential confounders, the HR and 95% CI of all-cause mortality for serum Tyr levels from the lowest to the highest quartile was 1.00 (reference), 1.31 (0.59-2.92), 2.17 (1.23-4.60), and 2.18 (1.01-4.71), respectively. 3 Compared with the traditional risk predictive model (C index = 0.773), adding serum Tyr levels increased the C index (C index = 0.787) and NRI (NRI = 0.267) for predicting all-cause mortality.

**Conclusions:**

Elevated serum tyrosine levels are independently associated with an increased risk of all-cause mortality, and may serve as a valuable biomarker for mortality risk prediction in Chinese older adults.

## 1 Introduction

Amino acids are the cornerstones of protein metabolism and play essential roles in synthesizing proteins, maintaining normal metabolism, converting neurotransmitters, regulating immunity, and providing energy (1). Abnormal amino acid metabolism leads to the development of metabolic diseases, including obesity, insulin resistance, and diabetes mellitus, and may also lead to cardiovascular diseases, immune system disorders, and cancers (2). Among many amino acids, a series of metabolomics and clinical studies focused on the link between branched-chain amino acids as well as aromatic amino acids and the development of metabolic and cardiovascular diseases (2–7). Previous research by our research group showed that branched-chain amino acids could increase the risk of metabolic diseases, including insulin resistance (8), newly-diagnosed type 2 diabetes (9), nonalcoholic fatty liver disease (10), dyslipidemia (11), and metabolic syndrome (12). Given their strong correlation with metabolic risk factors, elevated levels of branched-chain amino acids are indicative of an increased risk of developing cardiovascular diseases, including atherosclerotic heart disease, heart failure, and myocardial infarction, and suggest an unfavorable prognosis for cardiovascular diseases (7). Among aromatic amino acids, studies have demonstrated that elevated levels of phenylalanine (Phe) are linearly associated with 30-day mortality in patients experiencing stress in intensive care unit patients experiencing stress (13). Furthermore, chronic elevation of Phe levels has been found to correlate strongly with metabolic disorders (14), cardiovascular disease (7), and poor disease prognosis (15). However, the association of tyrosine (Tyr) with metabolic diseases, cardiovascular diseases, and all-cause mortality remains inconclusive and requires further investigation.

Tyr, a member of the aromatic amino acid family, plays a pivotal role in metabolism. Although the liver and kidneys can convert Phe to Tyr with the assistance of phenylalanine hydroxylase (16–18), this process is insufficient to meet the body’s normal requirements. In such cases, it is necessary to supplement the diet with additional Tyr sources. Tyr is metabolized via two principal pathways following its entry into the human body. The first is the generation of melanin and catecholamine hormones, and the second is the generation of acetoacetic acid and fumaric acid by enzymes involved in Tyr metabolism. These two compounds are involved in the tricarboxylic acid cycle and affect the metabolic processes of glycolipids (19). Concurrently, in the presence of intestinal flora, Tyr and its metabolites can also generate uremic toxins such as p-cresol, which are further absorbed into the serum by the intestine (20).

Prior studies have demonstrated a strong association of serum Tyr and metabolic disorders with a variety of diseases such as obesity (21), diabetes (22), cardiovascular disease (23), and cancer (24). However, evidence regarding the association between Tyr concentration and clinical prognosis, particularly all-cause mortality, remains limited and inconsistent. A cohort study from the United States and the European Union indicated that although serum Tyr was negatively associated with the incidence of major adverse cardiovascular events or mortality and no correlation between Tyr and all-cause mortality had been observed, the majority of Tyr metabolites produced by the gut microbiota had a positive association with major adverse cardiovascular events and all-cause mortality (25). These findings suggest that abnormal serum Tyr levels are significantly associated with mortality and may have potential value in clinical prognosis. Given this context, Tyr warrants attention due to its dual metabolic role as both an essential physiological substrate and a potential biomarker for adverse clinical outcomes.

Against the above background, Our study extends our team’s established research program on amino acid metabolism (branched-chain and aromatic amino acids), which has systematically established links between branched-chain amino acids and metabolic or cardiovascular disorders (8–12). We selected 1,238 subjects from the Diabetes Prevention Program of Huai’an City deeply investigate the potential correlation between branched-chain or aromatic amino acids with all-cause or cardiovascular causes of death over 9 years and further evaluated the predictive value of these amino acids for all-cause mortality in an elderly population.

## 2 Methods

### 2.1 Study population

**Figure 1:**
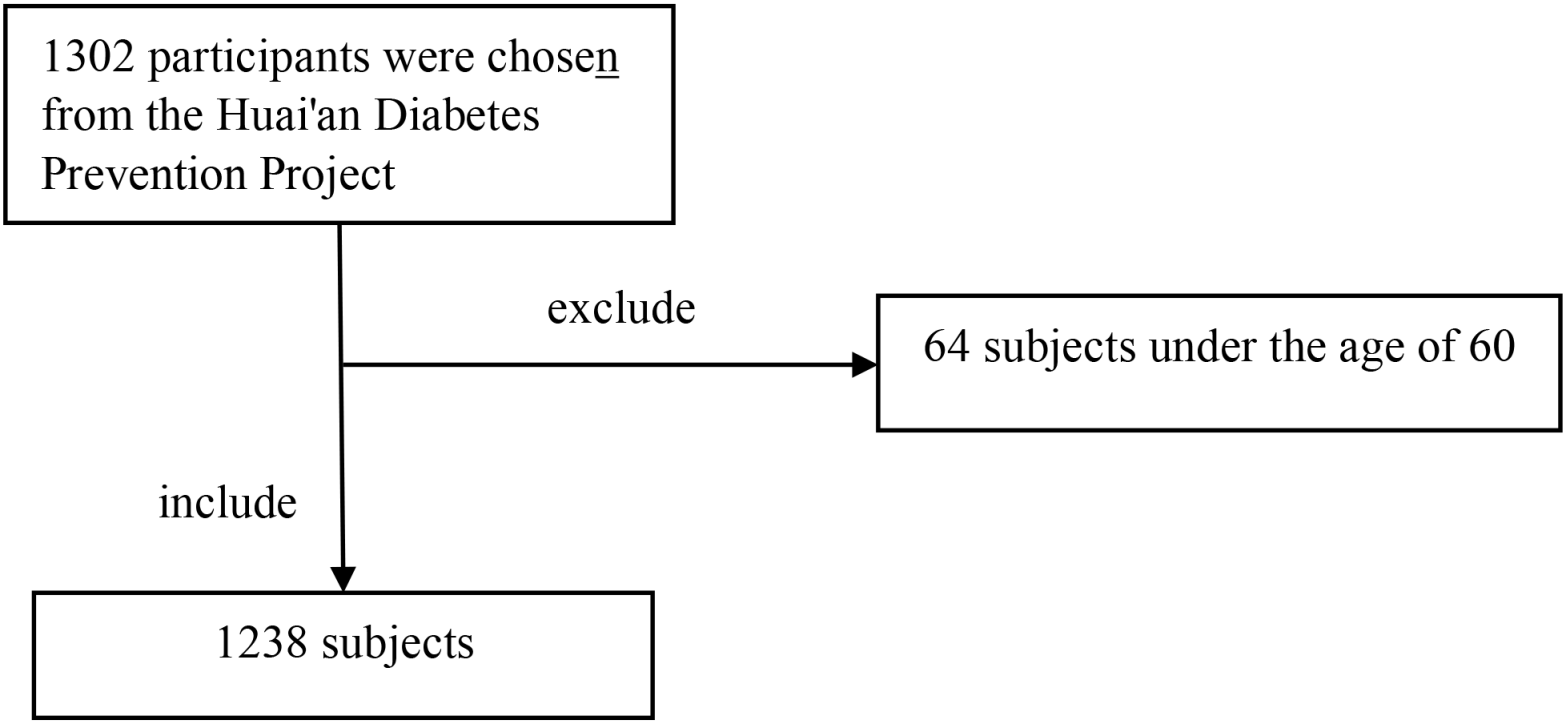
The procedure of subject selection.

In this study, 2,243 participants were enrolled in the Huai’an Diabetes Prevention Program (HADPP, ChiCTR-TRC-14005029) between August 2014 and September 2014. The initial inclusion and exclusion criteria have been described in our previous study (12). According to the World Health Organization study on population aging and human health (26), subjects aged ≥ 60 years were selected to observe further the associations of 5 amino acids at baseline with all-cause and cardiovascular mortality in the elderly population. Finally, 1,238 subjects were included in the study. **Figure 1** illustrates this procedure. At baseline, we collected demographic characteristics, lifestyle information, medical history, and fasting venous blood samples(from August to September 2014). With a follow-up period of 9 years until December 2023, we collected the subjects’ survival status, time of death, and cause of death from the Chinese Center for Disease Control and Prevention in Huai’an City.

This study was approved by the Ethics Committee of Huai’an Hospital, Affiliated with Xuzhou Medical University (Huai’an Second People’s Hospital). All the participants provided written informed consent.

### 2.2 Data collection at baseline

Baseline data, including demographics, lifestyle, and medical history, were systematically collected by trained investigators using a standardized questionnaire. Venous blood samples were collected at baseline between 7:00 AM and 9:00 AM after an 8-hour fast. The following laboratory indices were analyzed: fasting plasma glucose (FPG), hemoglobin A1c (HbA1c), triglycerides (TG), total cholesterol (TC), low-density lipoprotein cholesterol (LDL-C), high-density lipoprotein cholesterol (HDL-C), total bilirubin (TBIL), total protein (TP), alanine aminotransferase (ALT), aspartate aminotransferase (AST), creatinine (CREA) and blood urea nitrogen (BUN). Additionally, serum levels of leucine (Leu), isoleucine (Ile), valine (Val), phenylalanine (Phe), and tyrosine (Tyr) were quantified using the hydrophilic interaction chromatography-tandem mass spectrometry(HILIC-MS/MS) method, as previously described in our research (12).

### 2.3 Determination of follow-up mortality

All subjects were passively followed up during the 9-year follow-up period ending December 2023, and information on their survival status was obtained by contacting the Chinese Center for Disease Control and Prevention in Huai’an. The time and principal cause of death were ascertained by two experienced physicians from death certificates and autopsy reports. The principal causes of death were categorized according to the International Classification of Diseases, 10th Edition (ICD-10), with cardiovascular causes of death including heart disease (ICD-10: I00-I09, I11, I13, I20-I51) and cerebrovascular disease (ICD-10: I60-I69).

### 2.4 Clinical definition

Body mass index (BMI) was calculated as weight (kg) divided by height squared (m^2^). The estimated glomerular filtration rate (eGFR) was assessed from serum CREA levels according to the Chronic Kidney Disease Epidemiology Collaboration (CKD-EPI) formula (27). Hypertension was defined as a history of hypertension in medical records, a self-reported history of hypertension, resting systolic blood pressure (SBP) ≥140 mmHg, or diastolic blood pressure (DBP) ≥90 mmHg (28). According to the American Diabetes Association guidelines, prediabetes is defined as impaired fasting glucose (IFG), impaired glucose tolerance (IGT), or impaired HbA1c. Details are as follows (1): IFG: FPG level is 5.6-6.9 mmol/L; (2) IGT: blood glucose level is 7.8-11.0 mmol/L after a 2-hour oral glucose tolerance test; (3) impaired HbA1c: HbA1c level is 5.7%-6.4% (29). Diabetes mellitus is defined as follows: FPG ≥7.0 mmol/L, or 2-hour plasma glucose ≥11.1 mmol/L after administration of 75 g glucose, or the HbA1c concentration ≥6.5%, or history of diabetes mellitus in the medical record, or a self-reported history of diabetes mellitus (29). Dyslipidemia is defined as a plasma concentration of TC exceeding 6.20 mmol/L, TG exceeding 2.30 mmol/L, LDL-C exceeding 4.10 mmol/L, or HDL-C below 1.00 mmol/L (30).

### 2.5 Statistic analysis

Continuous variables were described as mean ± standard deviation for normally distributed data or interquartile range for non-normally distributed data, whereas categorical variables were expressed as percentages. Univariate Cox hazard analysis was used to compare the association of five amino acids (Leu, Ile, Val, Phe, and Tyr) with all-cause mortality risk. Considering non-cardiovascular causes deaths as competing risk events, the univariable Fine-Gray subdistribution hazard analysis was employed to evaluate the association between amino acids and cardiovascular mortality. Statistical significance was set at P <0.05.

To further analyze the association between Tyr and clinical parameters, subjects were grouped into quartiles according to serum Tyr levels (<13.58, 13.58-15.15, 15.15-16.97, and ≥16.97 μg/mL). Differences between groups were compared using one-way analysis of variance for continuous variables and the chi-square test or Fisher’s exact test for categorical variables. For the trend test, linear regression was selected for continuous variables for trend testing methods, and the trend chi-square test was chosen for categorical variables. Bonferroni correction was applied for key secondary analyses. The Spearman’s correlation analysis was used to calculate the association between the serum Tyr concentration and clinical indicators. Multivariable Cox proportional hazards regression was used to analyze the association between Tyr levels and all-cause mortality. Hazard ratios (HR) and 95% confidence intervals (95% CI) were reported using the lowest Tyr quartile as a reference group. Covariate selection via ridge regression identified sex, age, smoking status, and hypertension as stable predictors. Clinically relevant indicators of tyrosine metabolism (BMI, ALT, and eGFR) were additionally incorporated. Ultimately, the following potential confounding variables were adjusted: Model 1: age, sex, and BMI; Model 2: age, sex, BMI, ALT, eGFR, smoking status, and hypertension. We assessed the proportional hazards assumption for all Cox regression models using Schoenfeld residuals and log-log plots. Statistical analysis revealed no significant correlations between the residuals and time, confirming that the assumption was adequately satisfied for each Cox model in our study.

Harrell’s C-index and Net Weight Classification Improvement (NRI) were used to assess the value of adding Tyr to predict all-cause mortality. The reference model was constructed based on the current traditional risk factors, including sex, age, BMI, TG, TC/HDL ratio, HDL, HbA1c, smoking, history of diabetes, history of hypertension, and history of dyslipidemia (31).

In subgroup analyses, subjects were stratified by the following variables: gender (female or male), age (<70 or ≥70 years), BMI (<25 or ≥25 kg/m^2^), smoking (yes or no), hypertension status (yes or no), diabetes status (non-diabetic, prediabetic, or diabetic), ALT (<22 or ≥22 U/L), AST (<20 or ≥20 U/L) and eGFR (<89 or ≥89 ml/min/1.73m2). The hazard ratio (HR) and 95% confidence interval (CI) were calculated using Univariate Cox hazard analysis. Likelihood ratio tests were used to assess the interactions.

The data were analyzed using SPSS Statistics (Version 29.0.0.0, IBM, Chicago, IL, USA) and R studio (version 4.3.3). Two-sided p-values < 0.05 were considered statistically significant unless otherwise corrected.

## 3 Results

### 3.1 Association between 5 amino acids and all-cause or cardiovascular mortality

The study included 1,238 elderly participants (including 777 females and 461 males), with an average age of 69.8 ± 5.31 years and an average BMI of 24.62 ± 3.11 kg/m². At the 9-year follow-up, 1,169 subjects survived, and 69 subjects died, including 32 subjects who died of cardiovascular causes. A comparison of the clinical characteristics of the deceased and surviving groups is shown in **Supplementary Table 1**.

During the 9-year follow-up period, the all-cause and cardiovascular mortality rates were 6.11 and 2.83 per 1,000 person-years, respectively. The results of the univariate Cox hazard analysis for the associations of the 5 amino acids with all-cause mortality and the univariable Fine-Gray subdistribution hazard analysis for cardiovascular mortality are shown in **Table 1**. Only increasing plasma Tyr levels were associated with the risk of all-cause mortality (for each 1-μg/mL increase in Tyr, HR = 1.08, 95% CI = 1.01-1.17, p = 0.034). In contrast, Tyr was not associated with cardiovascular causes of death, and no associations of the remaining four amino acids with all-cause or cardiovascular mortality were observed.

**Table 1:** Association of 5 amino acids with all-cause or cardiovascular mortality.

Amino acids	All-cause mortality		CVD mortality	
	HR(95%CI)	*P* value	HR(95%CI)	*P* value
Leu	1.02 (0.98-1.06)	0.32	1.03 (0.97-1.09)	0.42
Ile	1.07 (0.99-1.15)	0.076	1.07 (0.97-1.18)	0.16
Val	1.03 (0.99-1.06)	0.20	1.04 (0.99-1.10)	0.14
Phe	1.02 (0.97-1.08)	0.46	0.99 (0.92-1.06)	0.68
Tyr	1.08 (1.01-1.17)	0.034	1.00 (0.90-1.10)	0.93

The associations between five amino acids and mortality outcomes were evaluated using univariable Cox proportional hazards regression for all-cause mortality and univariable Fine-Gray subdistribution hazards regression for cardiovascular mortality. *P*<0.05 was considered statistically significant. Leu, leucine; Ile, isoleucine; Val, valine; Phe, phenylalanine; Tyr, tyrosine; HR, hazard ratio; CI, confidence interval.

### 3.2 Baseline characteristics of the serum Tyr concentrations in quartiles

To understand the relationship between Tyr and all-cause mortality, we divided the participants into four groups according to the quartiles of serum Tyr concentrations and compared their clinical characteristics. As illustrated in **Table 2**, the clinical characteristics of sex, age, BMI, diabetes status, and FPG, TG, HDL-C, ALT, and AST levels significantly differed among the four groups (p < 0.05). As serum Tyr levels increased, the subjects were more likely to be older, have a higher BMI, and have a higher prevalence of dyslipidemia. Additionally, subjects with elevated Tyr levels exhibited higher TG, FPG, HbA1c, ALT, and AST levels and lower HDL-C levels(p for trend <0.05).

**Table 2:** Clinical and laboratory characters of Tyr quartiles grouping.

Characteristic	Tyr group				*P* value	*P* for trend
	Q1 (<13.58μg/mL, N=310)	Q2 (13.58-15.15, N = 309)	Q3 (15.15-16.97, N = 310)	Q4 (≥16.97μg/mL, N = 309)		
Female(%)	210 (67.7%)	179 (57.9%)	203 (65.5%)	185 (59.9%)	0.037	0.19
Age (years)	69.0 ± 5.4	69.5 ± 5.2	70.0 ± 5.4	70.6 ± 5.2	0.001	<0.001
BMI (kg/m^2^)	23.82 ± 3.05	24.43 ± 2.84	24.69 ± 2.91	25.55 ± 3.38	<0.001	<0.001
SBP (mmHg)	140 ± 19	140 ± 19	139 ± 20	140 ± 20	0.932	0.958
DBP (mmHg)	84 ± 12	84 ± 13	84 ± 14	84 ± 15	0.966	0.945
Smoke (%)	49 (15.8%)	60 (19.4%)	49 (15.8%)	56 (18.1%)	0.555	0.728
Drink (%)	47 (15.2%)	47 (15.2%)	45 (14.5%)	55 (17.8%)	0.686	0.435
Hypertension (%)	107 (34.5%)	104 (33.7%)	102 (32.9%)	118 (38.2%)	0.524	0.398
Diabetes(%)					0.006	0.073
Non-diabetes	225 (72.6%)	224 (72.5%)	216 (69.7%)	193 (62.5%)		
Pre-diabetes	34 (11.0%)	33 (10.7%)	40 (12.9%)	64 (20.7%)		
Diabetes	51 (16.5%)	52 (16.8%)	54 (17.4%)	52 (16.8%)		
Dyslipemia (%)	27 (8.7%)	32 (10.4%)	39 (12.6%)	48 (15.5%)	0.051	0.006
FPG (mmol/L)	5.79 ± 1.65	6.11 ± 2.00	6.09 ± 1.99	6.24 ± 1.87	0.026	0.006
HbA1c (%)	6.03 ± 1.14	6.08 ± 1.10	6.12 ± 1.05	6.21 ± 1.06	0.174	0.029
TG (mmol/L)	1.90 ± 1.26	1.95 ± 1.01	2.09 ± 1.16	2.32 ± 1.63	<0.001	<0.001
TC (mmol/L)	5.16 ± 0.90	5.21 ± 0.87	5.19 ± 0.91	5.18 ± 0.80	0.917	0.90
LDL-C (mmol/L)	2.69 ± 0.72	2.81 ± 0.71	2.77 ± 0.74	2.74 ± 0.65	0.199	0.57
HDL-C (mmol/L)	1.40 ± 0.41	1.39 ± 0.51	1.34 ± 0.49	1.29 ± 0.46	0.018	0.002
TBIL (μmol/l)	13.0 ± 7.6	12.6 ± 5.6	12.2 ± 4.9	13.0 ± 5.4	0.242	0.77
TP (g/L)	73.6 ± 3.9	74.0 ± 3.6	73.9 ± 3.8	74.1 ± 3.9	0.364	0.12
ALT (U/L)	22 ± 11	24 ± 11	24 ± 13	30 ± 16	<0.001	<0.001
AST (U/L)	21 ± 7	22 ± 7	22 ± 9	24 ± 10	<0.001	<0.001
CREA (μmol/l)	70 ± 16	71 ± 15	70 ± 14	73 ± 35	0.229	0.118
eGFR (ml/min/1.73m^2^)	83 ± 13	83 ± 11	82 ± 11	82 ± 12	0.405	0.144
BUN (mmol/l)	5.13 ± 1.40	5.26 ± 1.37	5.14 ± 1.26	5.22 ± 1.36	0.537	0.63
Tyr (μg/mL)	12.52 (11.55, 13.02)	14.40 (13.99,14.74)	15.91 (15.47, 16.46)	18.60 (17.66, 19.91)		

Continuous variables are described as mean ± standard deviation or interquartile range, and categorical variables are described as percentages (%). Differences between groups were compared using one-way ANOVA analysis for continuous variables, whereas the chi-square test, or Fisher’s exact test for categorical variables. The linear regression or the trend chi-square test was chosen to evaluate trends in baseline clinical parameters related to increasing Tyr levels. Bonferroni correction was applied for key secondary analyses. Two-sided p-values < 0.05 were considered statistically significant unless otherwise corrected. BMI, body mass index; SBP, systolic blood pressure; DBP, diastolic blood pressure; FPG, fasting plasma glucose; HbA1c, hemoHemoglobin A1c; TG, triglycerides; TC, total cholesterol; LDL-C, low-density lipoprotein cholesterol; HDL-C, high-density lipoprotein cholesterol; TBIL, total bilirubin; TP, total protein; ALT, alanine aminotransferase; AST, aspartate aminotransferase; CREA, creatinine; eGFR, estimated glomerular filtration rate; BUN, blood urea nitrogen.

### 3.3 The correlation between Tyr and clinical or laboratory parameters

Spearman’s correlation analysis revealed that baseline serum Tyr exhibited a significant positive correlation with age, BMI, FPG, HbA1c, TP, TG, ALT, and AST levels and a significant negative correlation with HDL-C (**Table 3**). Conversely, no correlations were observed between baseline serum Tyr levels and SBP, DBP, TC, LDL-C, TBIL, CREA, eGFR, or BUN.

**Table 3:** Spearman’s correlation analysis between Tyr and clinical or laboratory parameters.

Parameters	Correlation	*P* value
Age (years)	0.107	<0.001
BMI (kg/m^2^)	0.198	<0.001
SBP (mmHg)	0.000	0.998
DBP (mmHg)	0.009	0.757
FPG (mmol/L)	0.144	<0.001
HbA1c (%)	0.127	<0.001
TG (mmol/L)	0.190	<0.001
TC (mmol/L)	0.019	0.494
LDL-C (mmol/L)	0.026	0.352
HDL-C (mmol/L)	-0.140	<0.001
TBIL (μmol/l)	0.003	0.912
TP (g/L)	0.071	0.012
ALT (U/l)	0.199	<0.001
AST (U/l)	0.129	<0.001
CREA (μmol/l)	0.038	0.178
eGFR (ml/min/1.73m^2^)	-0.049	0.084
BUN (mmol/l)	0.037	0.197

Spearman’s correlation analysis was employed to assess the potential relationship between Tyr and clinical or laboratory parameters; p<0.05 was considered statistically significant. BMI, body mass index; SBP, systolic blood pressure; DBP, diastolic blood pressure; FPG, fasting plasma glucose; HbA1c, haemoHemoglobin A1c; TG, triglycerides; TC, total cholesterol; LDL-C, low-density lipoprotein cholesterol; HDL-C, high-density lipoprotein cholesterol; TBIL, total bilirubin; TP, total protein; ALT, alanine aminotransferase; AST, aspartate aminotransferase; CREA, creatinine; eGFR, estimated glomerular filtration rate; BUN, blood urea nitrogen.

### 3.4 Association of baseline serum Tyr with all-cause mortality

**Figure 2:**
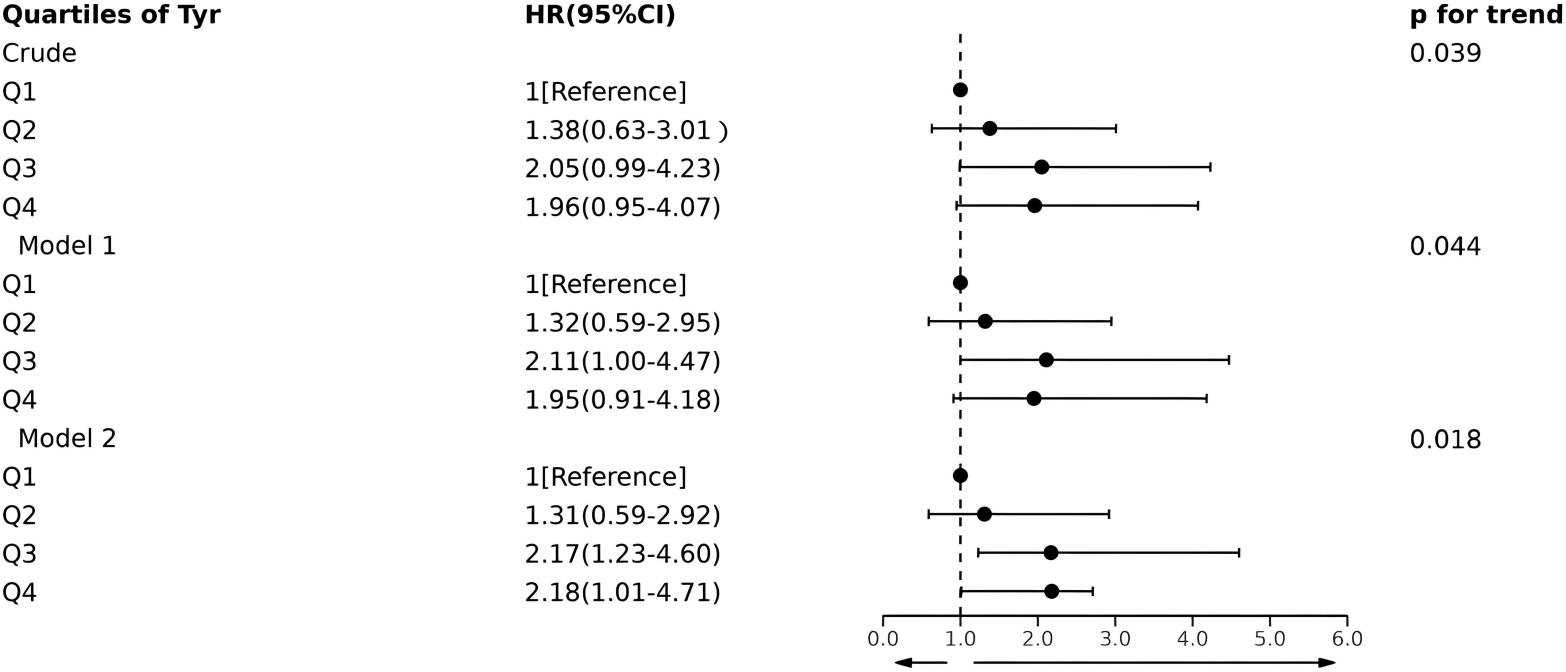
Forest plot of HR (95% CI) for Tyr quartile levels and risk of all-cause mortality. Model 1: adjusted for age, gender, and BMI; Model 2: additionally adjusted ALT, eGFR, hypertension and smoke. Tyr, tyrosine; HR, hazard ratio; CI, confidence interval.

The all-cause mortality rates per 1,000 person-years from the lowest to highest quartiles of serum Tyr levels were 3.84, 5.29, 7.85, and 7.53. **Figure 2** shows that multivariate Cox hazard analysis revealed an association between serum Tyr concentrations in the quartiles and all-cause mortality. The proportional hazards assumption for all Cox models was assessed using Schoenfeld residuals and log-log plots, with no significant violations detected. Although no association was identified between serum Tyr quartile groupings and all-cause mortality risk in the unadjusted or Model 1 analyses, adjusting for demographic characteristics and potential risk factors yielded a statistically significant association. The HR and 95%CI in the fully adjusted model from the lower to the upper quartiles for serum Tyr levels were 1.00 (reference), 1.31 (0.59-2.92), 2.17 (1.23-4.60), and 2.18 (1.01-4.71), respectively. The test for linear trends in Tyr quartiles was significant for both the unadjusted and adjusted models (p < 0.05).

### 3.5 Predictive value of baseline serum Tyr concentration

**Figure 3:**
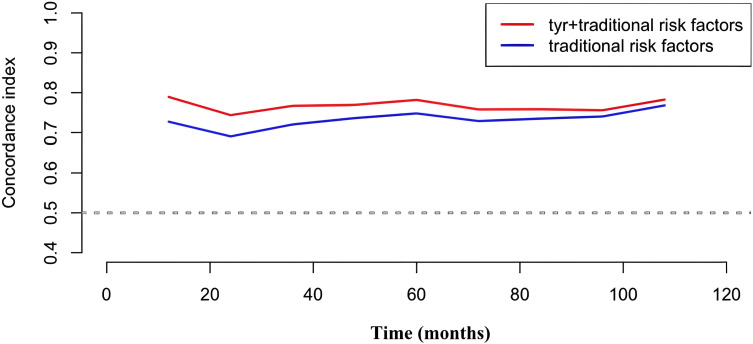
Point-in-time C-index plot depicting the C-index of the reference model with and without the additional inclusion of Tyr. Tyr, tyrosine.

The following traditional risk factors were selected to construct the reference prediction model: sex, age, BMI, TG level, TC/HDL cholesterol ratio, HDL cholesterol level, HbA1c level, smoking status, history of diabetes, history of hypertension, and history of dyslipidemia (31). The enhanced predictive efficacy of Tyr incorporation for all-cause mortality was ascertained. As shown in **Table 4**, further consideration of Tyr in the reference model revealed an increase in Harrell’s C-index from 0.773 (95% CI: 0.718-0.828) to 0.787 (95% CI: 0.734-0.840). Furthermore, the point-in-time C-index plot illustrated the predictive efficacy of all-cause mortality by incorporating additional Tyr into the reference model. (**Figure 3**). Additionally, the addition of Tyr to the reference model resulted in a notable improvement in the NRI (NRI=0.267, 95% CI: 0.0909-0.555) (**Table 4**).

**Table 4:** The predictive value of serum Tyr levels for all-cause mortality.

Model	C-index (95% CI)	NRI (95%CI)
Reference	0.773 (0.718-0.828)	–
Reference + Tyr	0.787 (0.734-0.840)	0.267 (0.0909-0.555)

Reference prediction model included gender, age, BMI, TG, TC/HDL ratio, HDL, HbA1c, smoking, history of diabetes, history of hypertension, and history of dyslipidemia. Tyr, tyrosine; NRI, Net Reclassification Improvement.

### 3.6 Subgroup analysis of baseline Tyr levels with all-cause mortality

**Figure 4:**
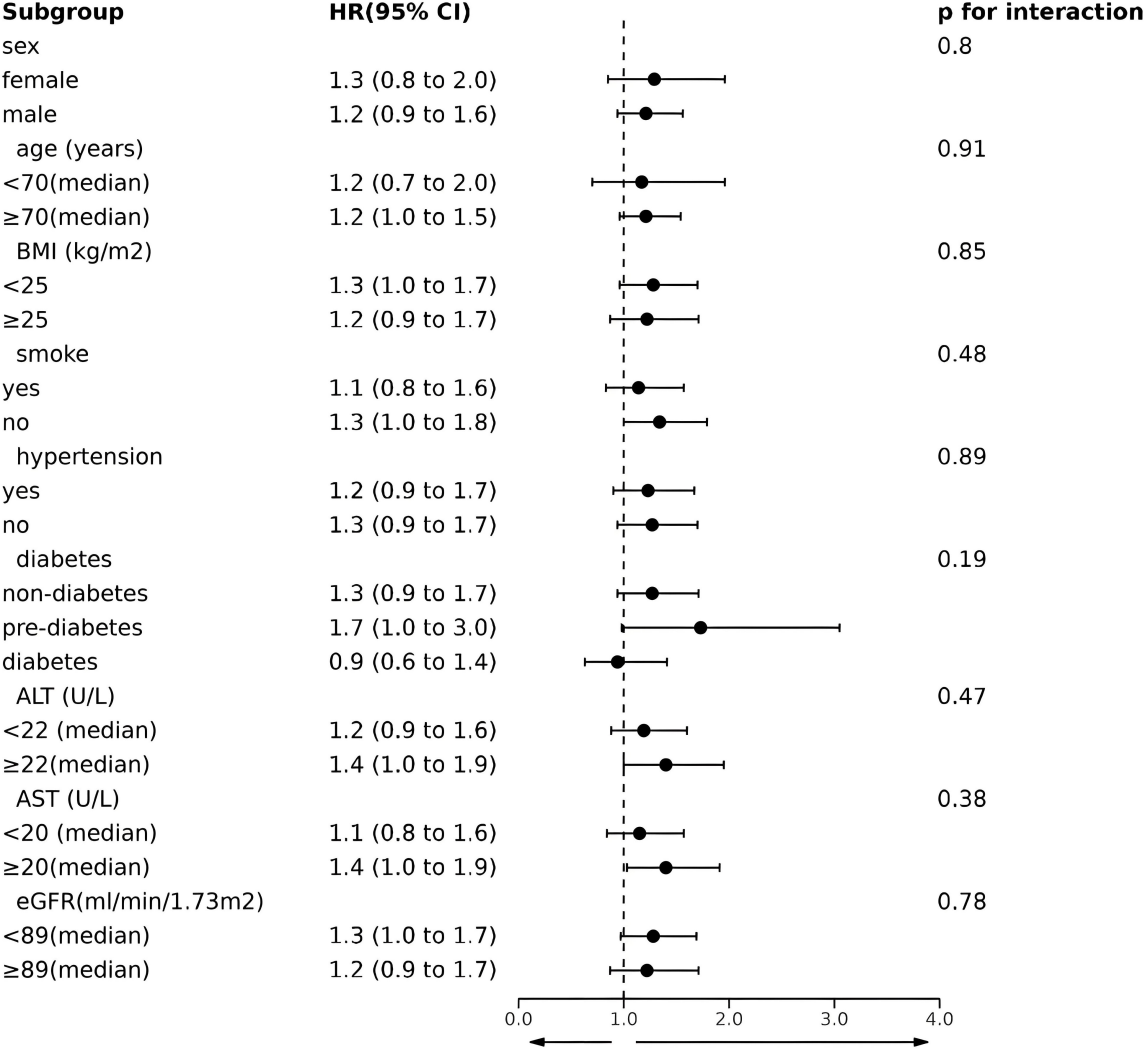
Forest plot for subgroup analysis on the association between Tyr and all-cause mortality. HR(95%CI) was calculated by univariate Cox hazard analysis. HR, hazard ratio; CI, confidence interval; BMI, body mass index; ALT, alanine aminotransferase; AST, aspartate aminotransferase; eGFR, estimated glomerular filtration rate.

Subgroup and interaction analyses were conducted to confirm the consistency of the relationship between serum Tyr concentrations and all-cause mortality across the cohort, and the results are presented in **Figure 4**. All subjects were stratified by gender (female or male), age (<70 or ≥70 years), BMI (<25 or ≥25 kg/m^2^), smoking (yes or no), hypertension status (yes or no), diabetes status (non-diabetic, prediabetic, or diabetic), ALT (<22 or ≥22 U/L), AST (<20 or ≥20 U/L) and eGFR (<89 or ≥89 ml/min/1.73m2). No significant interactions were detected between serum Tyr levels and the above stratification variables (p for interaction > 0.05).

## 4 Discussion

A growing body of evidence from recent years has indicated that metabolic disorders of amino acids are associated with adverse outcomes and an elevated risk of mortality in patients with metabolic or cardiovascular diseases (2), particularly branched-chain and aromatic amino acids (4, 6, 7, 32, 33). However, few studies have explored the association between branched-chain or aromatic amino acids and all-cause mortality. In this study, we investigated the relationship between the levels of five serum amino acids (Leu, Ile, Val, Phe, and Tyr) and all-cause or cardiovascular causes of death in a cohort of 1238 elderly subjects. The univariable survival analyses suggested that only serum Tyr levels were independently associated with all-cause mortality (for each 1 μg/mL increase in Tyr, HR = 1.08, 95% CI = 1.01-1.17, p = 0.034). After adjusting for potential confounding variables, the risk of all-cause mortality was 2.18 times higher for individuals with high quartiles of serum Tyr levels (≥16.97 μg/mL) compared to those with low quartiles of Tyr levels (<13.58 μg/mL). Moreover, the traditional risk prediction model incorporating serum Tyr levels enhanced the prediction of 9-year all-cause mortality. No correlations were identified between the remaining 4 amino acids and all-cause or cardiovascular mortality.

Only a few studies have identified a correlation between branched-chain or aromatic amino acids and all-cause and cardiovascular mortality (32–34), however, these findings remain controversial. A large metabolomics study indicated that while branched-chain amino acids and aromatic amino acids were positively associated with major adverse cardiovascular events in both the Finnish Institute for Health and Welfare (THL) and UK Biobank cohorts, their relationship with the risk of all-cause mortality remains controversial. In the THL Cohort, a negative correlation was observed between plasma aromatic amino acids and the risk of all-cause mortality; however, no such correlation was observed for branched-chain amino acids. In the UK BioBank Cohort, positive associations were observed between all-cause mortality and Phe, Leu, and Val levels, whereas no associations were identified between Tyr and Ile and all-cause mortality (35). It should be acknowledged that the subjects were younger than those in our study and of Finnish or British nationality. The present study suggested that among the five amino acids (Leu, Ile, Val, Phe, and Tyr), only serum Tyr was strongly associated with an elevated risk of all-cause mortality (for each 1 μg/mL increase in Tyr, HR = 1.08, 95% CI = 1.01-1.17, p = 0.034) (**Table 1**). This finding may be attributed to the fact that the majority of the subjects were elderly and in good condition, with no history of cardiovascular disease, cancer, or hepatic or renal dysfunction. Moreover, a previous cohort study of elderly community-dwelling men demonstrated the impact of frailty and age on the association between branched-chain amino acids and all-cause mortality. Following adjustment for age and frailty factors, branched-chain amino acids were not correlated with major adverse cardiovascular events or all-cause mortality. Furthermore, no correlation was observed between branched-chain amino acids and major adverse cardiovascular events or all-cause mortality in healthy elderly subjects (36). Thus, our findings suggest that aromatic amino acids, particularly Tyr, may be reliable indicators of all-cause mortality in healthy elderly individuals.

To further analyze the association between Tyr levels and all-cause mortality, we evaluated the correlation between serum Tyr levels and various clinical parameters. These findings indicate that elevated serum Tyr levels are associated with adverse clinical outcomes, including obesity (higher BMI), diabetes mellitus (higher FPG and HbA1c levels), dyslipidemia (higher TG levels and lower HDL-C levels), and abnormalities in liver function (higher ALT and AST levels). As metabolomic research has progressed, increasing evidence has indicated a correlation between aromatic amino acids and metabolic disorders, including obesity, diabetes, and dyslipidemia (3, 14, 37). In agreement with our study, a meta-analysis revealed that most amino acids, including Tyr, were elevated in individuals with obesity compared to those with normal weight (21). However, it remains unclear whether Tyr is a causative factor or a consequence of obesity. In our study, many subjects were obese at baseline (with a mean BMI of 24.62 ± 3.11 kg/m²), and the elevated Tyr levels may be linked to diminished amino acid catabolism in adipose tissue (38). Consistent with previous clinical studies, the present study indicated that elevated baseline Tyr levels are associated with poor glycemic control. Moreover, prior research has illustrated that high plasma Tyr levels not only predict an increased risk of pre-diabetes (39) but also indicate a heightened risk of developing type 2 diabetes mellitus (5) (22)and diabetic complications (15). In terms of lipids, a database from the Mexican Study of Childhood Obesity demonstrated that a serum amino acid profile consisting of arginine(Arg), Leu/Ile, Phe, Tyr, Val, and proline(Pro) was highly correlated with plasma TG and HDL-C levels and was predictive of future hypertriglyceridemia in obese children (3). The present study yielded analogous results, demonstrating an association between serum Tyr, TG, and HDL-C but not between TC and LDL-C levels. Some participants in the present study had a history of dyslipidemia and previous statin administration. The Statins may have resulted in a notable reduction in TC and LDL-C levels (40), which may have rendered the association of serum Tyr with LDL-C and TC levels insignificant. The association between Tyr and metabolic risk factors may further contribute to the development of cardiovascular and nonalcoholic fatty liver diseases.

Recently, the relationship between Tyr levels and liver function has been highlighted. In addition to a positive correlation between the incidence of hepatocellular carcinoma and mortality from chronic liver disease (41), Tyr levels also have a significant predictive value for the risk of death or liver transplantation in patients with liver cirrhosis (42). This is possible because 98% of Tyr is metabolized in the liver (43), therefore, when hepatocytes are severely damaged, Tyr metabolism is disrupted, resulting in an elevated level of Tyr (42). These findings are consistent with our findings that elevated serum Tyr levels can induce elevated ALT or AST levels. This may have led to the increased incidence of all-cause mortality.

Only a few studies have demonstrated a correlation between Tyr levels and all-cause or cause-specific mortality (25, 33, 44). The results of the present study indicate that, after adjusting for potential risk factors, the risk of all-cause mortality increased linearly with increasing serum Tyr levels. The risk of all-cause mortality was 2.18 times higher in the highest quartile of Tyr (≥16.97 μg/mL) compared to the lowest quartile of Tyr (<13.58 μg/mL). Interestingly, subgroup analyses revealed no statistically significant interactions between baseline serum Tyr levels and the following variables: age, sex, BMI, smoking status, hypertension status, diabetes status, ALT levels, AST levels, and eGFR levels. A dataset from the United States showed that a diet high in Tyr may decrease the risk of cardiovascular disease-related mortality independently, and the effect was more significant in non-Hispanic whites. However, no correlation was observed between tyrosinase intake and overall mortality (33). It is possible that the above study included subjects from the U.S. population of young adults, whereas our subjects were selected from the elderly Chinese population; therefore, the different findings may be related to factors such as age, race, and various dietary habits (45). There are some potential mechanisms through which Tyr may contribute to the increased risk of all-cause mortality: 1) Elevated plasma levels of amino acids, including Tyr, can inhibit glucose transport or phosphorylation, ultimately leading to insulin resistance in human skeletal muscle (46). Moreover, the terminal metabolites, Tyr, acetoacetic acid, and fumaric acid, can cause metabolic disorders and even death (19). 2) As Tyr levels increase, alterations in the synthesis of catecholamine hormones, another Tyr metabolite, may result in neurohormonal dysregulation, ultimately leading to an increased incidence of cardiovascular mortality (47). 3) Uremic toxins(e.g., p-cresyl sulfate and indoxyl sulfate), another Tyr metabolite produced by the intestinal flora, have been confirmed to elevate vasculogenic proinflammatory effects by recruiting leukocytes, resulting in vascular injury (48). Furthermore, these toxins increase the risk of major adverse cardiovascular events in individuals with impaired and preserved renal function (25).

This study revealed a notable advancement in Harrell’s C-index and NRI following the incorporation of serum Tyr levels into the reference model, which comprises traditional risk factors. This finding implies that Tyr level has a superior predictive effect on the risk of all-cause mortality in the elderly population. Only a few studies have suggested a predictive value of Tyr for mortality associated with acute heart failure (49) and cancer (50). Our results suggest the potential value of serum Tyr levels for clinical prognostic assessment and therapeutic guidance in the elderly. Furthermore, Tyr may serve as a helpful screening biomarker of poor prognosis in this population.

To our knowledge, this is the first prospective cohort study to reveal the association between Tyr and the risk of all-cause mortality in an Asian population using multifactorial COX regression analyses and subgroup analyses to remove the disturbance of potential confounders on the outcomes and to assess the independent variables more precisely. Additionally, this study demonstrated Tyr’s predictive capacity for the risk of all-cause mortality in an elderly Chinese population, which may provide some initial guidance into the potential relationship between Tyr and clinical prognosis to guide future studies. This study has some limitations. First, the number of all-cause mortality events in this study was relatively low (n = 69), which may be a consequence of the small sample size (n = 1238) and short follow-up period. Subsequently, we will extend the follow-up period and sample size to validate our findings. Second, this study was conducted at a single center and exclusively included elderly Chinese participants. Consequently, the results may not generalize to other ethnicities or younger populations. Third, the serum amino acid levels were measured only at baseline. Consequently, the potential influence of changes in any of the five amino acids during the follow-up period on these findings remains uncertain. Fifth, although the analysis was adjusted for demographic and traditional risk factors, residual confounding factors were not accounted for, including dietary protein or amino acid intake, markers of inflammation, frailty or sarcopenia, levels of physical activity or fitness and genetic predisposition. Due to the absence of these variables in our dataset, we were unable to adjust for them in the multivariable models. Future studies should aim to comprehensively collect and include these potential confounders to better elucidate the independent association and underlying mechanisms between serum tyrosine and mortality risk.

## 5 Conclusion

Our findings indicate that elevated serum Tyr levels are a potential risk factor and possess predictive value for the risk of all-cause mortality in the Chinese elderly population. These observations suggest the potential benefits of reducing serum Tyr levels in elderly populations.

## Data Availability

The raw data supporting the conclusions of this article will be made available by the authors, without undue reservation.
