# Supplementary material for: Serum tyrosine increases all-cause mortality in an older population
Source: Front Endocrinol (Lausanne). 2025 Jul 23;16:1552752. doi: 10.3389/fendo.2025.1552752 (PMC12325040; doi:10.3389/fendo.2025.1552752)
Supplement: Supplementary file 1 [file Table1.docx]

**supplementary materials**

| **Supplementary Table 1.** Comparison of the clinical characteristics of deceased and surviving groups | | | | |
| --- | --- | --- | --- | --- |
| **Characteristic** | **All**  **(N = 1,238)** | **Alive**  **(N = 1,169)** | **Dead**  **(N = 69)** | **p-value** |
| **Female(%)** | 777 (62.8%) | 759 (64.9%) | 18 (26.1%) | **<0.001** |
| **Age (years)** | 69.8 ± 5.3 | 69.6 ± 5.3 | 73.0 ± 4.9 | **<0.001** |
| **BMI (kg/m^2^)** | 24.62 ± 3.11 | 24.63 ± 3.12 | 24.50 ± 3.08 | 0.742 |
| **SBP (mmHg)** | 140 ± 19 | 140 ± 19 | 138 ± 20 | 0.385 |
| **DBP (mmHg)** | 84 ± 14 | 84 ± 14 | 83 ± 13 | 0.353 |
| **Smoke (%)** | 214 (17.3%) | 183 (15.7%) | 31 (44.9%) | **<0.001** |
| **Drink (%)** | 194 (15.7%) | 169 (14.5%) | 25 (36.2%) | **<0.001** |
| **Hypertension (%)** | 431 (34.8%) | 399 (34.1%) | 32 (46.4%) | **0.038** |
| **Diabetes(%)** |  |  |  | **0.006** |
| Non-diabetes | 858 (69.3%) | 822 (70.3%) | 36 (52.2%) |  |
| Pre-diabetes | 171 (13.8%) | 157 (13.4%) | 14 (20.3%) |  |
| Diabetes | 209 (16.9%) | 190 (16.3%) | 19 (27.5%) |  |
| **Dyslipemia (%)** | 146 (11.8%) | 138 (11.8%) | 8 (11.6%) | 0.958 |
| **FPG (mmol/L)** | 6.06 ± 1.89 | 6.00 ± 1.78 | 6.93 ± 3.04 | **0.015** |
| **HbA1c (%)** | 6.11 ± 1.09 | 6.08 ± 1.06 | 6.52 ± 1.38 | **0.012** |
| **TG (mmol/L)** | 2.07 ± 1.29 | 2.07 ± 1.31 | 1.91 ± 1.02 | 0.203 |
| **TC (mmol/L)** | 5.18 ± 0.87 | 5.20 ± 0.87 | 4.96 ± 0.87 | **0.029** |
| **LDL-C (mmol/L)** | 2.75 ± 0.70 | 2.76 ± 0.70 | 2.62 ± 0.76 | 0.129 |
| **HDL-C (mmol/L)** | 1.35 ± 0.47 | 1.35 ± 0.47 | 1.36 ± 0.46 | 0.946 |
| **TBIL (μmol/l)** | 12.7 ± 6.0 | 12.6 ± 5.9 | 14.1 ± 6.1 | 0.054 |
| **TP (g/L)** | 73.9 ± 3.8 | 74.0 ± 3.8 | 72.5 ± 4.5 | **0.007** |
| **ALT (U/l)** | 25 ± 13 | 25 ± 13 | 23 ± 13 | 0.197 |
| **AST (U/l)** | 22 ± 8 | 22 ± 8 | 22 ± 9 | 0.718 |
| **CREA (μmol/l)** | 71 ± 22 | 71 ± 22 | 77 ± 14 | **0.002** |
| **eGFR (ml/min/1.73m^2^)** | 82 ± 12 | 82 ± 12 | 80 ± 11 | 0.121 |
| **BUN (mmol/l)** | 5.19 ± 1.35 | 5.17 ± 1.34 | 5.59 ± 1.41 | **0.018** |
| Notes: Continuous variables are described as mean ± standard deviation or interquartile range, and categorical variables are described as percentages (%). Differences between groups were compared using one-way ANOVA analysis (continuous variables), and the chi-square test, or Fisher's exact test (categorical variables). p<0.5 was considered statistically significant and was highlighted in bold. BMI, body mass index; SBP, systolic blood pressure; DBP, diastolic blood pressure; FPG, fasting plasma glucose; HbA1c, haemoHemoglobin A1c; TG, triglycerides; TC, total cholesterol; LDL-C, low-density lipoprotein cholesterol; HDL-C, highdensity lipoprotein cholesterol;TBIL, total bilirubin; TP, total protein; ALT, alanine aminotransferase；AST, aspartate aminotransferase; CREA, creatinine; eGFR, estimated glomerular fltration rate; BUN, blood urea nitrogen. | | | | |
